# Current Perspectives on Development and Applications of Cocrystals in the Pharmaceutical and Medical Domain

**DOI:** 10.7759/cureus.70328

**Published:** 2024-09-27

**Authors:** Dhanashri D Chavan, Vandana M Thorat, Amol S Shete, Rohit R Bhosale, Sarika J Patil, Devkumar D Tiwari

**Affiliations:** 1 Department of Pharmacology, Krishna Institute of Medical Sciences, Krishna Vishwa Vidyapeeth (Deemed to be University), Karad, IND; 2 Department of Pharmaceutics, Krishna Institute of Pharmacy, Krishna Vishwa Vidyapeeth (Deemed to be University), Karad, IND; 3 Department of Pharmaceutics, Krishna Foundation’s Jaywant Institute of Pharmacy, Karad, IND

**Keywords:** active pharmaceutical ingredients, cocrystal formation, coformers, pharmaceutical and medical applications, pharmaceutical cocrystals

## Abstract

In recent years, the design of pharmaceutical cocrystals has garnered significant attention. The process of cocrystallization offers a remarkable opportunity to develop drug products with enhanced properties such as improved stability, solubility, hygroscopicity, dissolution rate, and bioavailability. This detailed review delves into this evolving area, thereby exploring its relevance in pharmaceutical formulation by defining cocrystals and their practical applications and also by discussing methods for their preparation as well as characterization. It also contrasts traditional and innovative techniques for cocrystal formation. Historically, cocrystals have been synthesized using methods like solvent evaporation, grinding, and slurry techniques; however, each has its own set of limitations under specific conditions. The latest trends in cocrystal formation lean toward more advanced approaches such as spray-drying, hot melt extrusion, and supercritical fluid technology, as well as the cutting-edge technique of laser irradiation. The aim behind developing new methods is not just to address the limitations of traditional cocrystallization techniques but also to streamline the process by introducing simpler steps and enabling a continuous production workflow for cocrystal products. In general, this full-length review article offers a report on various techniques available for the creation of pharmaceutical cocrystals, along with the methods for their evaluation. Moreover, it includes reporting developments and diverse applications of cocrystals along with the commercially available cocrystals in the pharmaceutical as well as medical domains.

## Introduction and background

The effectiveness and therapeutic action of drugs hinge significantly on their solubility and dissolution rates. Historically, drug discovery leaned on traditional medicines, but the landscape has shifted towards rational drug design and synthesis. This change has been propelled by the emergence of new diseases and advancements in drug development technologies. With a growing number of drug targets identified, researchers now utilize sophisticated methods such as high-throughput screening and combinatorial chemistry to create and assess potential drug candidates. However, as these lead compounds become larger and more lipophilic, they pose challenges related to solubility and permeability. Consequently, enhancing the solubility of active pharmaceutical ingredients (APIs) has become a focal point in contemporary drug development. Various strategies have been proposed to tackle solubility issues, including the formation of prodrugs, reducing particle size, creating salts, and utilizing solid dispersions, inclusion complexes, and polymorphs. Additionally, surfactants and multi-component molecular crystals have been explored, each with distinct advantages and challenges that depend on the physicochemical characteristics of the APIs and the excipients used. Physical modifications, such as increasing the surface area of particles, improving solubility, enhancing wetting properties, and stabilizing APIs, are commonly employed to optimize drug performance. In particular, multi-component crystals, encompassing hydrates, solvates, salts, and cocrystals, play a pivotal role in developing innovative solid forms in the pharmaceutical industry. For compounds that exhibit poor water solubility, several strategies can be adopted, including the formulation of amorphous solids, crystalline solid dispersions, or lipid-based systems. Among these techniques, cocrystallization has emerged as a particularly promising approach to addressing solubility challenges in drug development [1-4].

## Review

Cocrystals

As per the U.S. Food and Drug Administration (USFDA), cocrystals are crystalline substances comprising two or more distinct molecules, usually a drug and a cocrystal former (referred to as a "coformer"), integrated within the same crystal lattice. Pharmaceutical cocrystals have opened up new possibilities for engineering solid-state forms beyond the traditional forms of an active pharmaceutical ingredient (API), such as salts and polymorphs. These cocrystals provide a means to modify the physical characteristics of a drug, such as its solubility, while preserving its therapeutic effects. Moreover, cocrystals can also be specifically designed to improve stability as well as bioavailability of a drug, thereby enhancing the manufacturability of APIs during the production of drug products. Quinhydrone was the first synthesized cocrystal as documented in the literature, composed of a 1:1 hydroquinone and benzoquinone cocrystal [5,6].

Importance of Cocrystals

Cocrystals are significant because they can be engineered to exhibit superior physical properties compared to their individual starting materials. The enhancement of physical properties through cocrystal formation has been observed across various fields such as agrochemicals, pigments, solid explosives, and notably, pharmaceuticals, as most of the medications are administered in solid form. Physical characteristics of solids within a developed product directly influence its processing, delivery, and overall performance [7,8]. For instance, a drug's crystal structure plays a key role in determining its solubility in solution, which is essential for the drug's bioavailability in the body. Since approximately 80% of drugs are taken orally in solid form, considered a convenient and generally safe method, solubility becomes a critical factor. However, around 40% of these drugs face challenges due to low solubility. A major challenge in drug development is that approximately 80-90% of drug candidates face solubility problems, which significantly heightens the risk of failure in clinical trials [9,10]. Forming cocrystals with an appropriate coformer offers the possibility of enhancing solubility by altering the crystal structure, which can potentially increase the bioavailability of the compound. As research into cocrystals has grown, their application has broadened, allowing for the manipulation of various physical properties through cocrystal formation. Documented benefits include improvements in solubility, stability, dissolution rate, bioavailability, melting point, compressibility, friability, hygroscopicity, and bulk density [6,9]. Additionally, new uses are being explored, such as taste masking and extending intellectual property protections. The primary advantage of using cocrystals to modify drug properties is that the drug’s molecular structure remains unchanged, with the coformer being the component that alters properties [11,12]. Pharmaceutical cocrystals have introduced a unique perspective of solid-state modification, which the pharmaceutical industry is increasingly exploring. As research in cocrystal formation has progressed, numerous opportunities have emerged for manipulating physical properties through this approach. Over the past decade, interest in cocrystal structures and their applications has grown exponentially, as reflected by the significant increase in cocrystal structures recorded in the Cambridge Structural Database. The field has seen limited focus on the specific techniques used for cocrystallization. This review covers various methods commonly employed in cocrystal formation, including solid-state, mechanochemical, and liquid-assisted techniques. Furthermore, it explores several innovative approaches such as freeze-drying, microfluidic, and ultrasound-assisted cocrystallization, highlighting their potential in the synthesis of cocrystals [13-15].

Design of Cocrystals

Various theoretical approaches have been proposed to explain the mechanisms behind cocrystal formation, including hydrogen bonding propensity, analysis using the Cambridge Structural Database (CSD), supramolecular synthons, pKa values, and Hansen solubility parameters. Cocrystals based on principles of supramolecular synthesis represent a powerful method for discovering new pharmaceutical solid phases. These cocrystals combine multiple components in specific ratios where different molecular species interact through hydrogen bonding as well as non-hydrogen bonding. The formation of cocrystals can be better understood by considering the interaction of bond donors and acceptors in the materials being crystallized and how these interactions might occur [16]. Common functional groups involved in cocrystal formation include carboxylic acids, amides, and alcohols. In crystal engineering, homosynthons are typically formed between carboxylic acid dimers, amide dimers, and hydroxyl dimers, while heterosynthons form between carboxylic acid and amide groups, carboxylic acid and aromatic nitrogen/pyridine, hydroxyl and cyano groups, alcohol and ether groups, carboxylic acid and hydroxyl groups, and hydroxyl and aromatic nitrogen/pyridine in hydrogen bonding. Effective hydrogen bonding utilizes all good proton donors and acceptors. For designing cocrystals and selecting suitable coformers, understanding the supramolecular chemistry of functional groups in a molecule is essential, and hence, utilizing multiple theoretical approaches can enhance the selection process for potential coformers before conducting complex experimental screenings, resulting in superior efficiency in cocrystal screening [17,18].

Different strategies of cocrystals formation

Researchers have explored a variety of strategies for cocrystal preparation over time. Traditional methods such as solvent evaporation and grinding have been used, alongside solvent-based and solid-state techniques [19]. Different approaches such as crystallization techniques, suspension conversion, solvent evaporation, anti-solvent addition, and reaction crystallization have been employed. Recently, newer methods have emerged, such as ultrasound-assisted techniques, supercritical fluid atomization, spray-drying, and hot melt extrusion. Despite these advances, there remains a lack of standardization in the application and terminology of these methods. Variations in solvent selection, concentration of target molecules and coformers, equilibration times, and recovery processes are often not well documented. This inconsistency complicates efforts to replicate or compare methods, which can create confusion, particularly for newcomers to the field [20]. This review aims to systematically compile and describe the various cocrystal preparation techniques and their applications in one comprehensive resource. The goal is to standardize the information and consolidate progress in this rapidly developing area of research.

Solid-state methods

Solid-state grinding with references dating back to the late 19th century has been recognized for a long time. This method has gained significant traction in recent years due to its benefits, which include the simplicity and scalability of the process. Typical solid-state grinding techniques involve grinding solid mixtures, melt extrusion, and sonication, often performed at temperatures ranging from 80-85°C. In this approach, API and coformer are melted followed by mixing, subsequently leading to cocrystal formation at a precise stoichiometric ratio. While this method may not be suitable for thermally sensitive compounds, it is straightforward, scalable, and continuous [21].

Contact Formation

The formation of cocrystals can occur spontaneously by mixing pure API and coformer in a controlled atmospheric environment, without applying mechanical forces. However, pre-grinding the individual components before mixing has been reported to accelerate the cocrystallization process compared to using unmilled reactants [22].

Solid State Grinding

Solid-state grinding is a popular technique for producing cocrystal powders, typically executed in two main ways: neat (dry) grinding and liquid-assisted grinding. Neat grinding involves mixing solid materials in precise stoichiometric ratios, then grinding them together using a mortar and pestle, a ball mill, or a vibrational mill, usually for 30 to 60 minutes. This method offers several benefits, such as ease of use, rapid preparation, and the ability to produce a wide range of cocrystals. Dry grinding increases the specific surface area of the particles, enhancing intermolecular interactions and selectivity compared to dissolution-based cocrystallization methods. It is also useful for investigating hydrogen bond preferences. However, dry grinding can present challenges. Issues such as failure to form cocrystals, incomplete conversion, and the presence of crystalline defects or amorphous content may arise. Incomplete cocrystal formation can result in a mixture of cocrystals and unreacted starting materials, necessitating additional purification steps to achieve a pure cocrystal product [23].

Liquid-Assisted Grinding

This method involves incorporating a slight amount of solvent into the grinding process, thereby accelerating the kinetics of cocrystal formation. The solvent acts as a catalyst by either facilitating molecular diffusion or contributing to the formation of a multi-component inclusion framework, thereby enhancing supramolecular selectivity in the crystalline system. Notably, the solvent itself is not part of the final cocrystal product. The key benefits of this liquid-assisted grinding approach include improved performance, better control over polymorph production, and enhanced crystallinity of the final product. It enables the rapid formation of high-purity cocrystals and can be used to produce selective polymorphic forms with the ability to facilitate interconversion between crystalline forms based on the solvent’s polarity. However, this method does have its drawbacks. It is generally suitable only for small-scale applications, requires substantial energy, and may result in lower product purity compared to other methods [23].

Hot Melt Extrusion (HME)

HME is an advanced technique integrating the formation of cocrystal with the drug formulation process, consequently offering a streamlined approach to drug manufacturing. In HME, cocrystals are formed by heating the drug and coformers, which are mixed intensely to enhance surface contact without the need for solvents. The process involves heating to a specific temperature where the matrix material softens or melts, facilitating the cocrystal formation. Key requirements for the matrix material in HME include a low glass transition temperature (Tg) that is below the melting point of the cocrystal to allow for effective processing at lower temperatures, minimal noncovalent interactions with the drug or coformer to avoid interference, and a quick solidification step to ensure proper formation of the cocrystal. However, this method has limitations. Not all coformers and APIs are compatible with the molten matrix, and it is not suitable for unstable drugs [24].

Solution-based methods

Numerous methods are available for cocrystallization from solution, and these will be explored in detail. The fundamental driving force for crystallization is supersaturation. In a cocrystal system, there are two concentrations to consider: that of the target molecule and that of the coformer. The extent of supersaturation for cocrystallization is determined by the concentrations of both relative to the solubility of the cocrystal. In such systems, there are two key eutectic points: one where a mixture of the cocrystal and the target molecule is the stable solid phase, and another where a mixture of the cocrystal and coformer is stable. These eutectic points represent conditions of minimal solvent content, meaning that solubility is at its peak. Additionally, considering polymorphic compounds, those that can exist in multiple crystalline forms, can be beneficial. Such compounds exhibit structural flexibility, making them more amenable to forming different packing arrangements when combined with another molecule. This flexibility increases the likelihood of successful cocrystallization [25].

Slurry Crystallization

Slurry crystallization involves creating a suspension by mixing the active pharmaceutical ingredient (API) with suitable coformers and adding various solvents. After forming the suspension, the solvent is removed, followed by the drying of the solid material for five minutes under nitrogen flow. The resulting cocrystals are then characterized using powder X-ray diffraction (PXRD). This method is employed when the drug and coformer remain stable in the chosen solvent [15,26].

Evaporative Cocrystallization

Evaporative cocrystallization is a widely used technique for producing cocrystals, especially when single crystals are needed for diffraction analysis. In this method, stoichiometric quantities of the drug and coformer are dissolved in a common solvent, and to ensure the formation of clean cocrystals, it's crucial to separate individual crystals or bulk samples before the solvent evaporates completely. A slow evaporation rate is preferred to produce fewer, larger crystals rather than numerous smaller ones. Given that crystal structure identification is key in determining whether the crystals are cocrystals, salts, hydrates, or other polymorphic forms, evaporative cocrystallization is frequently referenced in cocrystal research. Ideally, cocrystallization should be conducted using three different solutions: one with a 1:1 stoichiometric ratio, one with excess coformer, and one with excess drug [26].

Cooling Crystallization

Although less commonly used, seeded cooling crystallization can be an effective method for cocrystal formation. This technique tends to be slower and more time-consuming compared to other methods. For instance, the cocrystal of darunavir and succinic acid demonstrated enhanced solubility, dissolution, and micrometric properties compared to darunavir alone. In another application, seeded cooling crystallization was employed to synthesize cocrystals of carbamazepine and nicotinamide from ethanol. This approach aimed to develop a scalable method for cocrystallization by carefully selecting solvents, identifying the thermodynamically stable cocrystal range, and managing desupersaturation kinetics throughout the process [25-27].

Anti-solvent method

This method is also called as vapor diffusion and is used to produce high-quality cocrystals by introducing an anti-solvent to a solution. This technique involves adding a solvent in which the target compound has low solubility to another solution where the compound is more soluble. This process promotes the precipitation of the cocrystals by creating supersaturation. The resulting mixture is filtered, and the solid product is then analyzed using X-ray powder diffraction (XRPD). Phase solubility diagrams are often constructed during these studies to determine the optimal solvent-to-anti-solvent ratio for effective cocrystal formation [27].

Crystallization by Reaction

This method is utilized to produce cocrystals rapidly, typically at constant temperature. In this process, nucleation and subsequent cocrystallization are governed by the solubility characteristics of the cocrystal components. Initially, the drug, being the less soluble component, is saturated in methanol and then filtered. The more soluble component, the coformer, is added in an amount just below its solubility limit. Due to the solubility constraints, the resulting cocrystals that precipitate are typically pure. Throughout the crystallization process, the concentrations of the components are monitored using high-performance liquid chromatography (HPLC) to ensure that the solid formed is indeed the cocrystal [28].

Ultrasound Aided Cocrystallization

Ultrasound-aided cocrystallization is also known as sonocrystallization and is employed in nano crystals synthesis. Drugs and coformers are dissolved in a suitable solvent, and cold water is circulated throughout the sonication process to maintain a consistent temperature within the sonicator and to prevent fragmentation. A suggested criterion for coformer material used here is that it should be capable of sublimation, which can facilitate the nucleation process through the vapor phase. This approach has been successful in producing pure cocrystals [24,28].

Spray Flash Evaporation Process

The spray flash evaporation process, initially developed for preparing semi-crystalline nanocomposites in explosives, leverages the flashing behavior of superheated liquids subjected to rapid pressure drops. This technique facilitates strong interactions between various drug-coformer pairs, resulting in accelerated crystallization rates. The process begins by dissolving the materials in a low-boiling solvent heated to around 60°C, followed by pressurization to 40-60 bars and subsequent atomization into a chamber through a heated hollow cone nozzle. Abrupt pressure drops cause superheated solutions to be thermodynamically unstable, subsequently causing excess energy to convert into latent energy, which in turn drives the cocrystallization of the compounds [24].

Supercritical Fluid Atomization Technique

This method utilizes the solvent capabilities of supercritical carbon dioxide (CO2) for suspending an API and coformer in a slurry within supercritical CO2. In this process, supercritical solutions rapidly expand when drug-coformer solutions in supercritical CO2 quickly depressurize (within 10-5 seconds) to atmospheric pressure. This rapid depressurization significantly reduces the solvent power of the fluid, causing a sharp increase in solute supersaturation. This sudden supersaturation triggers nucleation and crystallization, leading to the precipitation of fine particles. The technology offers the advantage of using non-toxic, highly volatile solvents that do not leave solvent residues in the final cocrystals [29].

Spray Drying

This method is a continuous and single-step technique that transforms liquids such as solutions, suspensions, or slurries into solid powders. The core principle involves converting a liquid feed into dried particles by spraying it through a hot gaseous medium. This method has been extensively utilized in various pharmaceutical applications, including the creation of micro- and nanoparticles for pulmonary delivery, solid dispersions, viral vectors, and pure drug particles. Its advantages stem from its continuous operation, high degree of control, and rapid processing. In the context of cocrystal production, spray drying offers the capability to embed cocrystals within an excipient matrix, improving the rheological properties of the resulting material. For systems where drugs and coformers have unequal solubility and pure cocrystals cannot be achieved through solvent evaporation, spray drying presents a viable alternative [30].

Miscellaneous techniques

Laser Irradiation

This technique uses a powerful CO2 laser for irradiating powder mixtures of coformers, prompting their transformation into cocrystal. Titapiwatanakun and colleagues applied this method to successfully synthesize caffeine cocrystals with oxalic acid and malonic acid, wherein their research indicates that for cocrystallization to proceed, the cocrystal components must experience considerable sublimation, consequently suggesting that the formation of the cocrystal likely involves molecular rearrangement and nucleation in the vapor phase [31].

Resonant Acoustic Mixing

This method has successfully produced various carbamazepine cocrystals using a resonant acoustic mixer operating at 80-100 G and 60 Hz. The resulting cocrystal products were isolated at different laboratory scales, including 100 mg, 1.5 g, and 22 g, indicating the technology's potential for scale-up [32].

Freeze Drying

Freeze drying, also known as lyophilization, is another technique employed for the formation of pharmaceutical cocrystals. While freeze drying is an established process widely used in biotechnology, pharmaceuticals, diagnostics, and the food industry, recent efforts have adapted it for cocrystallization. The freeze-drying process involves a multi-step operation where drying is achieved by first freezing the wet substance, followed by the sublimation of ice directly into vapor under low partial pressure of water vapor. This method, traditionally used to preserve various products, has also been demonstrated to be effective in preparing cocrystals [33].

Electrospraying

The electrospraying technique simultaneously generates and charges droplets using an electric field, wherein a high-potential capillary nozzle releases a solution of dissolved substances, and the electric field elongates the solution droplets, forming a jet that is subsequently dried, with the resulting particles collected on a charged powder collector [33].

Microfluidic and Jet Dispensing

This approach enables the dissolution of small amounts of parent compounds and coformers on a single chip through precise combinatorial mixing. In the microfluidic system, caffeine was introduced vertically and coformers horizontally, and the findings confirmed that screening for cocrystals using microfluidic chips is both consistent and reproducible [34].

Evaluation of cocrystals

Spectroscopic Analysis

Fourier-transform infrared spectroscopy (FTIR): FTIR is a widely adopted technique for predicting and determining the chemical conformation, intermolecular interactions, and compatibility between an API and its coformers. This technique allows for the analysis of the API, coformers, and resulting cocrystals over a wavelength range of 400-4000 cm-1. Known for its speed and non-destructive nature, FTIR is highly sensitive to changes in molecular structure and can detect functional groups effectively [32-34].

Terahertz time-domain spectroscopy (THz-TDS): THz-TDS, an analytical method used to characterize and identify cocrystals, is particularly effective in distinguishing between different supramolecular structures and identifying chiral and racemic molecules within a sample [32-34].

Solid-state nuclear magnetic resonance (NMR): Solid-state NMR is frequently employed to characterize and identify salts and cocrystals while evaluating the structure through the detection of local conformational changes and hydrogen bonds. The underlying principle of this technique involves nuclear shifts induced by irradiation, which distinguish the cocrystal from excipients [32-34].

Thermal Gravimetric Analysis (TGA)

TGA is useful for determining the weight change of a sample under the influence of temperature over time. Differential scanning calorimetry (DSC) is often used in conjunction with TGA to determine cocrystal formation. TGA is instrumental in identifying the precise drying temperature as well as the various reaction steps involved in a compound. TGA also identifies the presence of hydrated or solvated crystal forms and volatile components and analyzes the decomposition or sublimation of cocrystals. TGA helps predict crystal purity, solvated/hydrated forms, and the thermal stability and compatibility of cocrystals [34].

Hansen solubility study

The Hansen solubility parameter is a key tool for predicting the miscibility of a drug and its coformer in a cocrystal formation and also helps to predict the compatibility of pharmaceutical materials, wherein cohesion energy associated with the Hansen parameter is used to predict physicochemical properties like melting point and solubility [8,32].

Dissolution study

In-vitro dissolution studies are conducted to evaluate the dissolution efficiency of formulated drugs using appropriate dissolution apparatus and media as specified by official compendia. Samples are collected at defined intervals and analyzed via HPLC or ultraviolet (UV) spectrophotometry [33,34].

Stability study

Stability is a critical parameter in evaluating cocrystals, providing insights into how different climatic storage conditions and environmental factors, such as humidity, light, and temperature, affect the shelf life of a drug or drug product. Stability studies under specific temperature and humidity conditions are performed over predetermined time intervals to assess the shelf life of cocrystal products under various storage scenarios [35].

Applications of cocrystals

Over the last twenty years, the exploration of pharmaceutical cocrystals has garnered significant attention from both academic researchers and the pharmaceutical industry [36,37]. This surge in interest has led to an abundance of studies focusing on both the theoretical foundations and practical applications of cocrystallization. Notably, some cocrystals have progressed from research to clinical use, with examples like escitalopram oxalate-oxalic acid (Lexapro®), sacubitril-disodium valsartan-water (Entresto^TM^), ertugliflozin-L-pyroglutamic acid, and tramadol-celecoxib either available on the market or undergoing clinical trials [38]. Cocrystallization presents a compelling approach for enhancing the physicochemical properties of an active pharmaceutical ingredient (API) without altering the drug’s molecular structure. This method is particularly effective in addressing the challenges associated with the poor solubility and bioavailability of drugs belonging to BCS class II and IV, which constitute approximately 70% of molecules in drug development [39]. Beyond improving dissolution and bioavailability, cocrystallization has also been successfully employed to enhance chemical stability, reduce hygroscopicity, and improve the mechanical and flow characteristics of APIs [36,37]. Additionally, this technique offers potential benefits in the purification of compounds and the separation of enantiomers [40]. Machado Cruz and his research team have developed a new cocrystal, composed of an antifungal compound itraconazole possessing poor water solubility, to form itraconazole-terephthalic acid cocrystal. Their comprehensive study investigated the solid-state properties and formation of this cocrystal, and remarkably, the cocrystal demonstrated stability in water. Their findings indicated that, unlike other itraconazole cocrystals studied previously, there is a clear connection between the intrinsic and powder dissolution rates and solubility of the coformer used, and furthermore, the team examined how physical mixtures of this cocrystal with various commonly used excipients influenced its dissolution behavior [41].

In a separate investigation, Nugrahani and colleagues explored the synthesis of diclofenac sodium-L-proline cocrystals in both mono- and tetrahydrate forms, wherein they employed single-crystal X-ray diffraction to thoroughly analyze these hydrates. The study found that these hydrated forms exhibited superior solubility and faster dissolution rates compared to both the diclofenac sodium salt and the anhydrous diclofenac acid-L-proline cocrystal. Remarkably, the salt cocrystal reverted into a physical blend of diclofenac acid and L-proline after drying, a transition that was surprisingly reversible, and also under conditions of 72% relative humidity at 25°C, the diclofenac sodium-L-proline tetrahydrate reformed from the dried mixture [42]. In another significant contribution, Buol et al. reported the first-ever cocrystals of nefiracetam, a drug known for its nootropic properties. Through an extensive cocrystal screening process involving 133 potential coformers and utilizing liquid-assisted grinding, they identified 13 fresh cocrystals subsequently characterized by single-crystal X-ray diffraction. This outcome demonstrated the ability to manipulate solid-state properties, such as melting points, by varying the coformer. Three of these cocrystals, formed with biocompatible coformers, underwent further scrutiny. The research also delved into the solubility and dissolution characteristics of these newly identified cocrystals [43].

Kale and colleagues investigated how cocrystallization impacts the tabletability of rivaroxaban, discovering that the tabletability of the rivaroxaban-malonic acid cocrystal is notably superior to that of pure rivaroxaban or malonic acid alone. The order of tabletability of malonic acid < rivaroxaban < rivaroxaban-malonic acid can be explained by examining the crystal packing. Factors such as the presence or absence of slip planes, the topology of these slip planes, the extent of intermolecular interactions, and d-spacing all play crucial roles. Rivaroxaban's crystal structure includes slip planes with both flat-layered and zigzag-layered topologies, and this study's findings illuminate how crystals with multiple slip-plane systems undergo deformation [44]. In another study, Wroblewska et al. presented a novel coformer, 1-hydroxy-4,5-dimethyl-imidazole 3 oxide, and explored its cocrystallization with barbituric and thiobarbituric acid using high-resolution solid-state NMR during ball-milling. The study assessed how different polymorphic and tautomeric forms of barbituric/thiobarbituric acid influence cocrystallization, where the resulting cocrystals at concentrations up to 100 µM demonstrated no cytotoxicity in HeLa and 293T cells, suggesting that 1-hydroxy-4,5-dimethyl-imidazole 3 oxide is biocompatible. However, despite these promising biocompatibility results, the coformer did not significantly enhance solubility [45].

Salas-Zuniga and their team investigated the impact of hydroxypropyl methylcellulose (HPMC) and methylcellulose on the dissolution characteristics of two cocrystals of nitazoxanide. They formulated polymer-based powders using nitazoxanide-succinic acid and observed a significant enhancement in the apparent solubility of nitazoxanide compared to formulations containing only the native API. This increase in solubility was likely attributable to the polymers' ability to delay nucleation and inhibit crystal growth [46]. Witika et al. reported on the creation of a cocrystal combining lamivudine and zidovudine. They characterized this dual-drug cocrystal using various techniques such as Raman spectroscopy, X-ray powder diffraction, FT-IR spectroscopy, energy-dispersive X-ray spectroscopy, and differential scanning calorimetry. The research also explored the application of surfactants to produce and stabilize nano-cocrystals [47]. Following this, the team utilized a Design of Experiments (DoE) approach to refine the cold sonochemical synthesis of lamivudine-zidovudine nano-cocrystals by incorporating various surfactants and polymers. The resulting nano-cocrystals demonstrated reduced cytotoxicity in HeLa cells compared to a physical blend of two APIs [48].

Although numerous cocrystallization techniques have been developed, only a few are suitable for large-scale production. Among these scalable methods are spray drying, spray congealing, and hot melt extrusion (HME) [49]. Successfully implementing these techniques in an industrial setting demands a deep understanding of the underlying principles, careful control of process parameters to ensure high yield and product quality, and awareness of how excipients might influence cocrystal composition during manufacturing. Spray congealing, a relatively recent addition to the cocrystallization toolkit, involves passing a molten mixture of the API and coformer through an atomizer. Duarte et al. demonstrated the potential of spray congealing in cocrystal production, successfully generating caffeine:salicylic acid and carbamazepine:nicotinamide cocrystals using this technique. Their work also highlighted the ability to manipulate the properties of caffeine:glutaric acid cocrystals by fine-tuning process parameters, such as those related to atomization and cooling [50]. Spray drying, a solvent-based cocrystallization technique, has been effectively used by Urano et al. to create cilostazol cocrystals with three different coformers: 2,4-dihydroxybenzoic acid, 4-hydroxybenzoic acid, and 2,5-dihydroxybenzoic acid. Their research showed that the cocrystals produced via spray drying were comparable in quality to those obtained from other cocrystallization methods. Moreover, the cilostazol cocrystals generated by spray drying exhibited enhanced dissolution behavior compared to those made using alternative techniques, reinforcing the scalability of spray drying for cocrystal production [51].

In another investigation, Walsh and colleagues assessed the performance of spray drying versus hot melt extrusion for creating ibuprofen and isonicotinamide cocrystals, including various excipients in the process. Their findings indicated that spray drying was particularly effective, consistently yielding high-quality cocrystals even with the inclusion of excipients. Similarly, Patil et al. developed successfully cocrystals of carbamazepine:nicotinamide using spray drying and found that the developed cocrystals were of comparable quality to those produced via liquid-assisted grinding (LAG). This research supports the use of spray drying as a viable method for the industrial production of pharmaceutical cocrystals, especially given its simplicity, scalability, and cost-effectiveness. However, the technique does require large quantities of hazardous organic solvents, which limits its classification as a green technology [52]. On the other hand, HME offers a solvent-free alternative for cocrystallization. Jafari et al. explored HME for manufacturing ibuprofen:isonicotinamide cocrystals and achieved complete conversion of the raw materials into the cocrystal by optimizing temperature and screw speed parameters. Moreover, they investigated the role of Soluplus (polymeric excipient), thereby finding that it lowered the cocrystallization temperature and enhanced the mechanical properties of the cocrystals [53]. Additionally, it has been demonstrated that using screws designed for intensive mixing and kneading can further improve cocrystal yield [54]. Butreddy and colleagues achieved the preparation of cocrystals of aripiprazole:adipic acid through HME with a 5% Soluplus, leading to cocrystals with significantly improved solubility and dissolution characteristics [55].

Commercially available cocrystals

The effective integration of cocrystallization in the pharmaceutical sector is evident on the basis of the approval and market presence of several cocrystal-based drugs. Among these include Suglat®, Entresto®, and Steglatro®, each featuring cocrystal-based active pharmaceutical ingredients (APIs). Suglat®, developed by Astellas Pharma and Kotobuki Pharmaceutical and approved in Japan in 2014, is used to treat diabetes. Its API, ipragliflozin, is a sodium-glucose cotransporter 2 inhibitor that tends to absorb water nonstoichiometrically, leading to hydrate formation during storage. However, cocrystallization with L-proline stabilizes ipragliflozin, preventing this undesired conversion. In some cases, cocrystal forms of drugs were developed or identified after the drug had already been approved. For instance, valproic acid, a medication used to treat epilepsy, is available as sodium valproate (both acid and sodium salt), wherein the acid form is liquid at room temperature and the sodium salt is highly hygroscopic. The cocrystal form combining valproic acid:sodium valproate in a 1:1 ratio is less hygroscopic than the individual components and is commercially known under names like Depakote®, Epilim, and divalproex sodium. Another example is Escitalopram oxalate (Lexapro®), which was later identified as a cocrystal. Similarly, chloral betaine (Beta-chlor®), a sedative, was recognized as a cocrystal at a later stage, consisting of chloral hydrate and betaine, with the cocrystal formation enhancing the thermal stability of chloral [56]. Entresto®, a drug-drug cocrystal developed by Novartis, is designed to reduce the risk of heart failure. It combines valsartan and sacubitril in a fixed-dose combination wherein the crystal structure consists of anionic forms of valsartan, sacubitril, sodium cations, and water molecules in a molar ratio of 1:1:3:2.5, along with additional excipients, as studied by Feng et al. [57]. Steglatro® for type-2 diabetes mellitus contains ertugliflozin as an API and L-pyroglutamic acid as a coformer. Ertugliflozin is naturally an unstable amorphous material, but its stability and physicochemical properties are significantly enhanced through cocrystallization with L-pyroglutamic acid in a 1:1 ratio [58]. Various methods can be employed for cocrystal screening, with solvent-based cocrystallization presenting several challenges. These include selecting an appropriate solvent, managing differences in API and coformer solubility within that solvent (congruent versus incongruent solubility), and determining optimal heating and cooling profiles. Solid-state grinding offers a preferable alternative for cocrystal screening, though it can occasionally induce phase transformations in pharmaceutical cocrystals [59,60]. The synthesis and characterization of cocrystals are not without challenges. Issues such as the formation of salts, solvates, or hybrids, as well as the inherent instability of cocrystals, especially in solution, pose significant obstacles. Moreover, cocrystals may revert to a less soluble parent drug form in solution, and polymorphism further complicates the development process [61,62].

Challenges associated in the development of cocrystals

A significant hurdle in the creation of pharmaceutical cocrystals is the challenge of selecting appropriate conformers. Given the vast number of potential conformers, there’s a pressing need for robust screening tools that can accurately predict which conformers are likely to form cocrystals. Once potential candidates are identified, they must then undergo experimental evaluation to confirm cocrystal formation. In recent years, research into cocrystals has surged, leading to the accumulation of valuable data that can aid in predicting conformer viability. Several methods have emerged as effective for this purpose, including assessing hydrogen bond propensity, utilizing the Cambridge Structural Database, employing the supramolecular synthon approach, applying the pKa rule, and considering the Hansen solubility parameters. Despite these advancements, the development of more refined screening tools remains essential for enhancing the practical application of cocrystallization within the pharmaceutical sector. Synthesizing and characterizing cocrystals comes with several significant challenges. These include the potential formation of salts, solvates, or hybrid structures, as well as the inherent instability of cocrystals themselves. In solution, cocrystals may exhibit varying stability, dissolution profiles, and solubility depending on factors like pH, ion concentration, surfactant levels, and the possibility of reverting to less soluble parent drug forms. Additionally, polymorphism can further complicate cocrystal behavior. Key issues that arise during cocrystal preparation include the risk of dissociation due to interactions with excipients, the potential for conformers to be replaced by formulation components, alterations in cocrystal stoichiometry, and the likelihood of converting to a less soluble parent drug during dissolution. When it comes to scaling up cocrystal production, achieving consistent control over stoichiometry is crucial. Furthermore, the lack of robust in vitro-in vivo correlations for cocrystals can hinder development timelines. Despite these hurdles, the presence of a limited number of cocrystal-based drugs on the market suggests that overcoming these challenges is indeed possible [22,58].

Future perspectives

Cocrystallization presents a compelling alternative to traditional approaches like salt formation, solvates, and polymorphs for enhancing the physicochemical properties and processability of active pharmaceutical ingredients (APIs). The cocrystallization technique acts as a boon for the BCS Class II drugs, belonging to the category of low solubility and high permeability. The limited availability of solvates and polymorphs, along with the absence of suitable ionizable groups in certain APIs, restricts the utility of these conventional methods in developing pharmaceutically acceptable forms. Over the past decade, significant research advancements have been made in various aspects of cocrystallization, including screening techniques, characterization methods, production processes, and formulation strategies. Regulatory bodies such as the USFDA and European Medicines Agency (EMA) have acknowledged the important role of pharmaceutical cocrystals in advancing drug candidates with improved properties. However, cocrystallization has not yet become a standard practice in pharmaceutical production. To integrate cocrystals more widely in the industry, it is crucial to focus on standardizing their synthesis, optimizing the parameters that affect cocrystal quality, such as purity, yield, and reproducibility, and ensuring that cocrystal screening and development are integral parts of the drug development pipeline [58].

## Conclusions

Pharmaceutical cocrystals represent an innovative category of substances that offer the potential to enhance key physical properties, resulting in new, stable, and patentable solid forms. These multi-component crystalline structures can significantly improve crucial physicochemical characteristics, such as solubility, dissolution rate, and chemical and physical stability. As a result, they often exhibit superior properties compared to the unaltered drug. In the last ten years, substantial research efforts have propelled progress in multiple areas of cocrystallization. This includes advancements in screening and developing cocrystals, enhancing characterization methods, refining production techniques, and optimizing formulation strategies. To fully integrate cocrystals into the pharmaceutical industry, future research should focus on standardizing synthetic methods, identifying and optimizing factors that impact cocrystal quality (such as purity, yield, and reproducibility), and incorporating cocrystal screening and development into the drug development pipeline. 
